# The effect of soil pollution with petroleum-derived substances on *Porcellio scaber* Latr. (Crustacea, Isopoda)

**DOI:** 10.1007/s10661-018-7181-6

**Published:** 2018-12-28

**Authors:** J. Gospodarek, P. Petryszak, H. Kołoczek, M. Rusin

**Affiliations:** 1 Department of Agricultural Environment Protection, University of Agriculture in Krakow, Al. Mickiewicza 21, 31-120 Kraków, Poland; 2 Unit of Biochemistry, Institute of Plant Biology and Biotechnology, Faculty of Biotechnology and Horticulture, University of Agriculture in Krakow, al. 29-Listopada 54, 31-425 Kraków, Poland; 3 Department of Inorganic Technology and Environmental Biotechnology, Cracow University of Technology, ul. Warszawska 24, 31-155 Kraków, Poland

**Keywords:** Polycyclic aromatic hydrocarbons, Oil contamination, Life parameters, Isopods, Total petroleum hydrocarbon depletion

## Abstract

Presented research aimed at investigating the effect of short-term contact with petroleum-derived substances (PDSs) on life parameters of *Porcellio scaber* Latr. (Isopoda) and accumulation of polycyclic aromatic hydrocarbons (PAHs) in its body. The influence of presence of *P. scaber* on the total petroleum hydrocarbons (TPH) content in soil was also determined. The following objects were established: control—unpolluted soil; soil polluted with petrol; soil polluted with diesel fuel and soil polluted with used engine oil. Every pollutant was used in the amounts equal to 6000 mg of fuel/kg d.m. of soil 15 months earlier. In the laboratory, survival and body mass change of *P. scaber* reared in investigated soils were observed. The delivered food was not contaminated with PDSs. *P. scaber* reveals a considerable resistance in a short (4 weeks) contact with PDSs, evidenced as high survivability (from 68% on the soil polluted with engine oil to 77% on the soil polluted with diesel fuel) and undisturbed increase in body mass (on the level similar to control). It indicates the potential usefulness of this animal as a monitoring organism. No positive correlation was observed between TPH depletion in the soils contaminated with PDSs and *P. scaber* presence during 4 weeks of the experiment. PAH level in *P. scaber* bodies was generally very low (with the highest level of anthracene 0.40 μg/g of wet mass—after 4 weeks of rearing on the diesel fuel–contaminated soil), which may confirm the thesis about considerable abilities of isopods for biotransformation of these pollutants and low susceptibility to these xenobiotic penetration through integuments. However, a tendency for accumulation for phenanthrene and anthracene in conditions of soil polluted with diesel fuel was observed respectively 0.07 and 0.21 μg/g of wet mass for phenanthrene and 0.22 and 0.40 μg/g of wet mass for anthracene, observed successively in the 2nd and 4th week of rearing.

## Introduction

Microorganisms, mainly bacteria such as *Pseudomonas*, *Moraxella*, *Alcaligenes*, *Oligella*, *Acinetobacter*, *Methylobacterium*, *Stenotrophomonas*, *Morganella*, and *Bacillus*, play a crucial role in a breakdown process of soil polluting petroleum-derived substances (PDSs) (Kaszycki et al. 2010, 2011). The presence of macroinvertebrates may increase the rate of PDS decomposition, as has been demonstrated on an example of earthworms *Lumbricus terrestris* (Schaefer and Filser 2007), *Dendrobaena veneta* (Hickman and Reid 2008a, b), or *Eisenia fetida* (Schaefer et al. 2005; Tejada and Masciandaro 2011). *Porcellio scaber* Latr. is one of the commonest representatives of terrestrial crustaceans. Its role in the process of plant material decomposition was thoroughly examined on an example of various types of forest litter (Kautz and Topp 2000). It was determined that its presence contributes to increase in soil microorganism biomass and microbial respiration. It was also demonstrated that availability of macroelements, such as C_org_, N_tot_, P, K, Mg, and Ca was increasing in the presence of *P. scaber*, although the intensity of this process depended on the kind of litter, the degree of its degradation, and soil type (Van Wensem et al. 1993; Kayang et al. 1994; Kautz and Topp 2000). The abovementioned process is connected with mechanical crushing of plant material, owing to which it is more easily mixed with soil particles, but also with release of specific chemical substances. It was also proved that isopods prefer litter colonized by microorganisms. A feedback between the presence of microorganisms and crustaceans was observed. Isopods feeding contributes to an increase in microorganism biomass but the presence of microorganisms causes that this food is more eagerly selected by these animals (Uesbeck and Topp 1995). Moreover, terrestrial isopods (especially *Porcellio scaber*) appeared to be very suitable indicators of heavy metals bioavailable fraction in polluted soil or leaf litter (Gal et al. 2008; Udovic et al. 2009; Udovic and McBride 2012). Their abundance was also a parameter investigated in an assessment of soil contamination with petrochemical wastes (Faulkner and Lochmiller 2000). The available literature provide information on the effect of selected elements of PDSs such as f. ex. benzo[*a*]pyrene on life parameters and growth of these animals (Van Straalen and Verweij 1991; Van Brummelen and Stuijfzand 1993). However, there is no information about the influence of PDSs (which are mixtures of many different components) in realistic environmental conditions on life traits of isopods or on their potential role in remediation of contaminated soils. The features, such as survival or growth are regarded as most sensitive indicators of the degree of soil pollution with PAHs, for earthworms, *Collembola* or *Gastropods* (Eom et al. 2007).

Any animal can play an important role in the clean-up of contaminated soil if it is not too sensitive to the pollutants. Therefore, it is crucial to determine the effect of analyzed type of pollution on its life parameters. Some dangerous components of oil derivative pollutants, e.g., polycyclic aromatic hydrocarbons, may accumulate in animal bodies and therefore pose a hazard for the successive links of food chain. According to the literature, isopods are exposed to PAHs mainly owing to consumed food (Van Brummelen et al. 1996). They reveal abilities for metabolizing some of these compounds, as has been demonstrated on an example of pyrene (Stroomberg et al. 1999, 2003, 2004a, b; Luthe et al. 2002) or for bioaccumulation (Broerse et al. 2012; Kampe and Schlechtriem 2016). However, soil contamination with petroleum products usually occurs locally, on limited (sometimes small) areas, so exposition of isopods to contaminants through their food intake may be rare. Because of that, it is worth investigating how sensitive are isopods to xenobiotic penetration through their integuments.

The main goals of the research were (a) testing the effect of PDSs (petrol, diesel fuel, and used engine oil) present in soil (not in food) on *P. scaber* life parameters, such as survival rate and change in body mass; (b) determining the influence of *P. scaber* presence on the total petroleum hydrocarbons (TPH) content in contaminated soil; (c) assessment of the effect of PDSs in soil on accumulation of polycyclic aromatic hydrocarbons (PAHs) in these animals’ bodies. We sought to find out whether *P. scaber* presence might accelerate the remediation of petroleum-contaminated soil, as well as evaluate the potential usefulness of this animal as a bioindicator or monitoring organism for the assessment of PDSs presence in the soil.

## Material and methods

The research started in 2009 in the field conditions at the Experimental Station of the University of Agriculture in Mydlniki near Krakow (Poland; 50.0815° N, 19.84730° E). In autumn 2009 indigenous soil (loamy-sand) was placed in 1 m^3^ containers with maintained natural layers arrangement. The containers were then dug into the ground, so that their upper area was on the same level with the surrounding soil. The soil in containers was left untouched for 8 months to allow it to restore its natural biological efficiency. Detailed characteristics of the soil, the containers and its arrangement in the experimental field were given in a previous paper (Gospodarek et al. 2016). In June 2010, the soil in containers was polluted (by pouring) with the following PDSs: petrol (BP Unleaded 95), diesel fuel (BP Diesel Fuel), and used engine oil (PLATINUM Classic Semisynthetic 10 W-40, used for 1 year in a petrol engine). Detailed characteristics of used petroleum products were given in our earlier paper (Rusin et al. 2017). Every pollutant was used in the amounts equal to 6000 mg of fuel kg^−1^ d.m. of soil. Four objects were identified: (1) control—unpolluted soil, (2) soil with simulated petrol leak (P), (3) soil with simulated diesel fuel leak (DF), and (4) soil with simulated used engine oil leak (EO). The whole experiment was conducted in four replications according to randomized block design. During the next 15 months, the pollutants undergo natural decomposition. Rearing of *P. scaber* was conducted in September 2011 (i.e., 15 months after soil pollution) on the soil collected from individual containers. The animals were obtained in the vicinity of the containers’ locality. The rearing was conducted for 4 weeks in constant conditions, at the temperature of 24 °C in 500 cm^3^ containers. Twenty-one *P. scaber* specimens (the same body mass) were placed in each container with 300 g of soil. The containers were covered with a gauze for better ventilation. Finely cut carrot pieces were the food supplied every week. Constant soil moisture was maintained by means of distilled water sprinkling. The number of live specimen and their body mass were registered each week of rearing. The experiment was conducted in four replications. Soil collected from individual containers and kept without *P. scaber* was treated the same way as soil with animals to evaluate the effect of *P. scaber* presence on TPH content in the soil.

The analysis of TPH content in the soil was made thrice: in the beginning of the experiment, after 2 and 4 weeks of rearing. The analysis of PAH content in *P. scaber* bodies was made twice: after 2 and 4 weeks of rearing.

### Analysis of TPH content in the soil

#### Assessment of soil dry mass

In order to determine the soil dry mass, crucibles were dried by c.a. 12 h at 105 °C and then cooled in the desiccator for 1 h. Dried and cooled crucibles were weighed on electronic scales with accuracy to 1 × 10^−3^ g and then 10 g of soil was placed in them and afterwards they were weighed once more. The crucibles were put into a thermostat for 5 h, then again cooled in the desiccator for 1 h and weighed. On the basis of obtained results a percentage of dry mass was determined. The assessments were made in 2 independent replications and the obtained final result was an average value.

#### Preparation of soil samples for analysis

About 10–11 g portions of soil were weighed into plastic containers (120 cm^3^). Subsequently, 1 cm^3^ of 18% hydrochloric acid was added to the weighed soil. The samples were mixed intensively using glass rods. In order to improve the penetration of light petroleum, water was removed from the soil samples by adding c.a. 12.5 g of calcinated anhydrous magnesium sulphate (VI) (72 h, 105 °C), and then the samples were intensively mixed. The samples prepared in this way were sealed tightly and left for c.a. 12 h to dry up. Prior to the extraction the soil samples were transferred quantitatively to extraction thimbles (Cellulose Extraction Thimbles, GRADE 603; Sigma-Aldrich, Cat. #: Z612456).

#### Assessment of oil derivative content in the samples using gravimetric method

The assessments were made using gravimetric method. 300 cm^3^ Erlenmeyer flasks were dried for 12 h in a thermostat (at 105 °C) and then cooled in the desiccator for 1 h, and weighed on an analytical balance with the accuracy to 1 × 10^−5^ g. When the weighing was complete, the flasks were coupled with Soxhlet apparatus and reflux condensers. Extraction thimbles containing previously prepared soil samples were placed in the Soxhlet apparatus. 230 cm^3^ of light petroleum (Petroleum Benzine Boiling Range 40–60; Sigma-Aldrich, Cat. #: 32299) was supplied to the extraction system. The extraction involving multiple rinsing of soil samples with organic solvent was conducted at the temperature of 70–80 °C for 6 h. Afterwards, the extracts were concentrated to obtain post-extraction remains on the bottom of Erlenmeyer flasks. The flasks were transferred to the thermostat and dried at 105 °C for 1.5 h, carried to the desiccator and cooled for 1 h. After cooling, the flasks were weighed on an analytical balance. On the basis of previously obtained results and determined dry matter content in soil, the concentration of oil derivatives was calculated. The assessments were made in two independent replications and the obtained final result is their average.

### Analysis of PAH content in animal material

#### Preparation of hexane extracts from animal material

In order to obtain hexane extract, between 0.8 and 1.0 g of animal material was weighed into glass tubes. Subsequently 1 cm^3^ of 1-methylchrysene solution in acetone (50 μg/cm^3^) was added, to serve as an internal standard and 10 cm^3^ of hexane. Homogenization of the animal material was conducted in a laboratory homogenizer (10,000 rpm). The extraction comprised 3 homogenization cycles, lasting 1 min each. When the homogenization cycle was completed, the obtained hexane extract was decanted, whereas 10 cm^3^ of the solvent was again added to the remaining animal material. In order to obtain clear supernatants, the hexane extracts were centrifuged (3000 rpm; 4 °C, 10 min) and then transferred quantitatively to Erlenmeyer flasks. The centrifugal thimbles were washed with hexane, which was added to previously decanted extracts. The extracts obtained in this way were condensed in a vacuum evaporator and the remains were suspended in 1 cm^3^ of hexane. The prepared samples were then left for chromatographic analyses.

#### Chromatographic analyses

##### Analytical standards

During the chromatographic analyses, a commercial set of PAH standards (Supelco 525 PAH Mix AQ, cat. # 48953-U) containing acenaphthylene, fluorene, phenanthrene, anthracene, pyrene, benz[*a*]anthracene, chrysene, benzo[*b*]fluoranthene, benzo[*k*]fluoranthene, benzo[*a*]pyrene, dibenz[*a*,*h*]anthracene, benzo[*ghi*]perylene, and indeno[*1,2,3-cd*]pyrene was used. The qualitative analysis was based on retention times and mass spectra of individual hydrocarbons; the quantitative analysis was conducted on the basis of calibration curves prepared within the range of concentrations from 0.5 to 50 μg/cm^3^ individual for respective compounds.

##### Gas chromatography

Chromatographic analysis of polycyclic aromatic hydrocarbons contained in the animal material were conducted using Shimadzu GC-17A ver.3 gas chromatograph equipped with Shimadzu QP-5000 mass spectrometer. Chromatographic separations were conducted in SLB-5 ms capillary column (Supelco, 60 m × 0.25 mm × 0.25 μm). The following temperature program was set for the column: 50 °C (hold for 2 min), and 5 °C/min up to 330 °C (hold for 12 min). The injector and linker temperatures were 335 °C and 330 °C, respectively. The total separation time was 70 min. Linear velocity of carrier gas (helium 5.0) was 25 cm/min. The 1-μL samples were dosed automatically using Shimadzu AOCi-20 autoinjector operating in splitless mode with sampling time set to 1 min. Detection of individual analytes was conducted as selected ion monitoring (SIM) using 64 mass ions characteristic for the assessed hydrocarbons. The analysis of obtained chromatographic data was conducted using GCMS-Solution Ver.1.2 software (Shimadzu Corporation, Japan).

### Statistical analysis

The obtained results were analyzed, checked for normality (Shapiro–Wilk test with Lilliefors correction) and equality of variance (Levene’s test). The significance of differences between the means were tested by two-factor variance analysis (STATISTICA 13.1 software), and the means were differentiated by Fisher’s LSD test at *p* < 0.05.

## Results and discussion

**Fig. 1:**
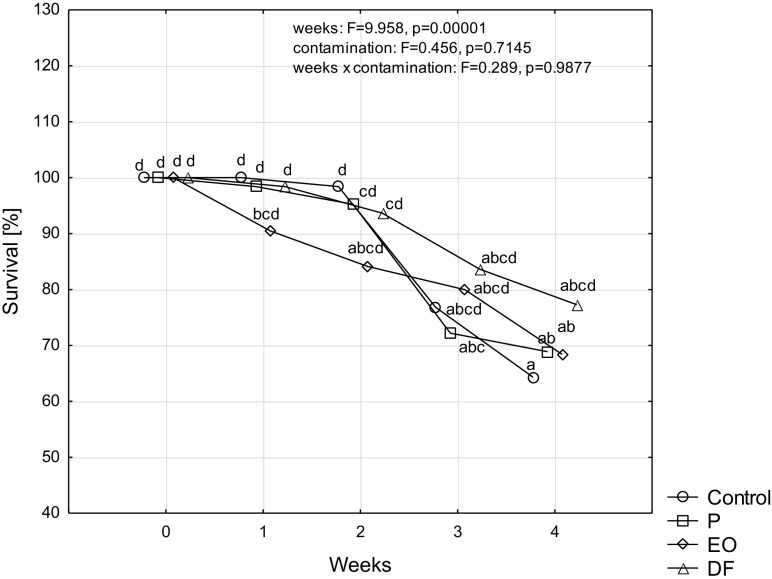
Survival of *Porcelio scaber* L. [%] rearing on soil contaminated with petroleum products: unleaded petrol (P), used engine oil (EO), and diesel fuel (DF) during four weeks of experiment (100% equals to 84 animals provided at the beginning of the experiment in each experimental group). Means marked with the same letters do not differ significantly according to LSD test at *p* < 0.05. Factors: weeks × contamination

Almost all analyzed cultured groups revealed a similar lethality during rearing (Fig. 1). After 4 weeks the percentage of live specimens fluctuated from 64% on the control soil to 77% on the soil polluted with diesel fuel. The earliest deaths, already after 1 week of rearing, were registered among the specimens kept on the soil contaminated with engine oil (c.a. 10% of the specimens from the initial group died). Also in the 2nd week of rearing the highest lethality persisted in the object with engine oil polluted soil. However, starting from the 3rd week, also specimens in the other research groups started to die, including the control. At that time, the lowest number of specimens died in the soil polluted with diesel fuel.

**Fig. 2:**
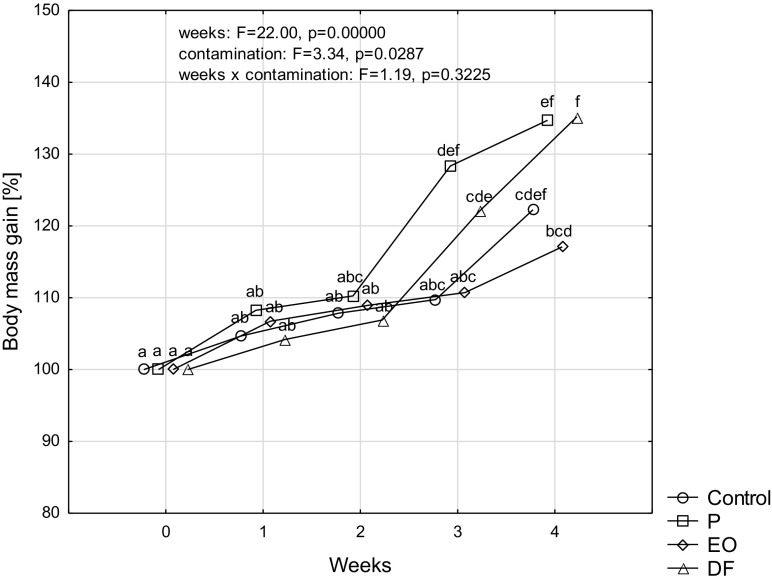
Body mass gain of a single individual of *Porcelio scaber* L. [%] (100% equals to the body mass at the beginning of the experiment in each experimental group) rearing on soil contaminated with petroleum products: unleaded petrol (P), used engine oil (EO), and diesel fuel (DF) during 4 weeks of experiment. Means marked with the same letters do not differ significantly according to LSD test at *p* < 0.05. Factors: weeks × contamination

After 4 weeks of rearing all test groups were characterized by a significant increase in an average body mass of single specimens (Fig. 2). Among the test groups with the oil derivative-polluted soil a relatively weakest increase in body mass was noted when *P. scaber* had contact with used engine oil, since statistically significant increase in body mass, in comparison with the initial group (by c.a. 17%) was registered only in the 4th week of rearing. In case of the objects where the soil was polluted with petrol or diesel fuel, the significant increase in body mass in relation to the initial group (respectively by 28% and 22%) was observed in the 3rd week of rearing. For the abovementioned objects, the average increases in body mass of single specimens in the 4th week of rearing were approximate and reached 35% in comparison to the start of rearing, whereas the increase in body mass in control specimens after 4 weeks of rearing was 22%.

Research on aquatic invertebrates points higher resistance of isopods than amphipods to PDSs pollution. They are also classified to the organisms quite easily recovered after oil spill (Lecklin et al. 2011). Growth of isopods, such as *Oniscus asellus* or *Porcellio scaber*, was limited only when their diets contained 100 μg/g or more of benzo[*a*]pyrene (B[*a*]P). On the other hand, no effect of B[*a*]P was observed at the concentration of 31.6 μg/g (Van Straalen and Verweij 1991; Van Brummelen and Stuijfzand 1993). At 10-fold higher concentration of B[*a*]P (316 μg/g), growth of *O. asellus* was reduced by 58%, while *P. scaber* by 26%. The authors explained the differences in response to the presence of pollutants, by the difference in body mass of the specimens of both species—the body mass of a single *P. scaber* specimen was about 3-fold higher than *O. asselus*. General survival rate of *P. scaber* in the abovementioned experiments was 76%, i.e., it was very similar to observed in the presented experiments. The great resistance of *P. scaber* to petroleum products was confirmed also by Faulkner and Lochmiller (2000). The densities of terrestrial isopod populations in their research were even 180-fold greater on sites contaminated with petrochemical wastes than on reference sites, although usually most of epigeic and edaphic invertebrates reacts with a decline in their density in the presence of oil-derived pollutants (Gospodarek 2008; Gospodarek et al. 2016; Rusin and Gospodarek 2016).

**Fig. 3:**
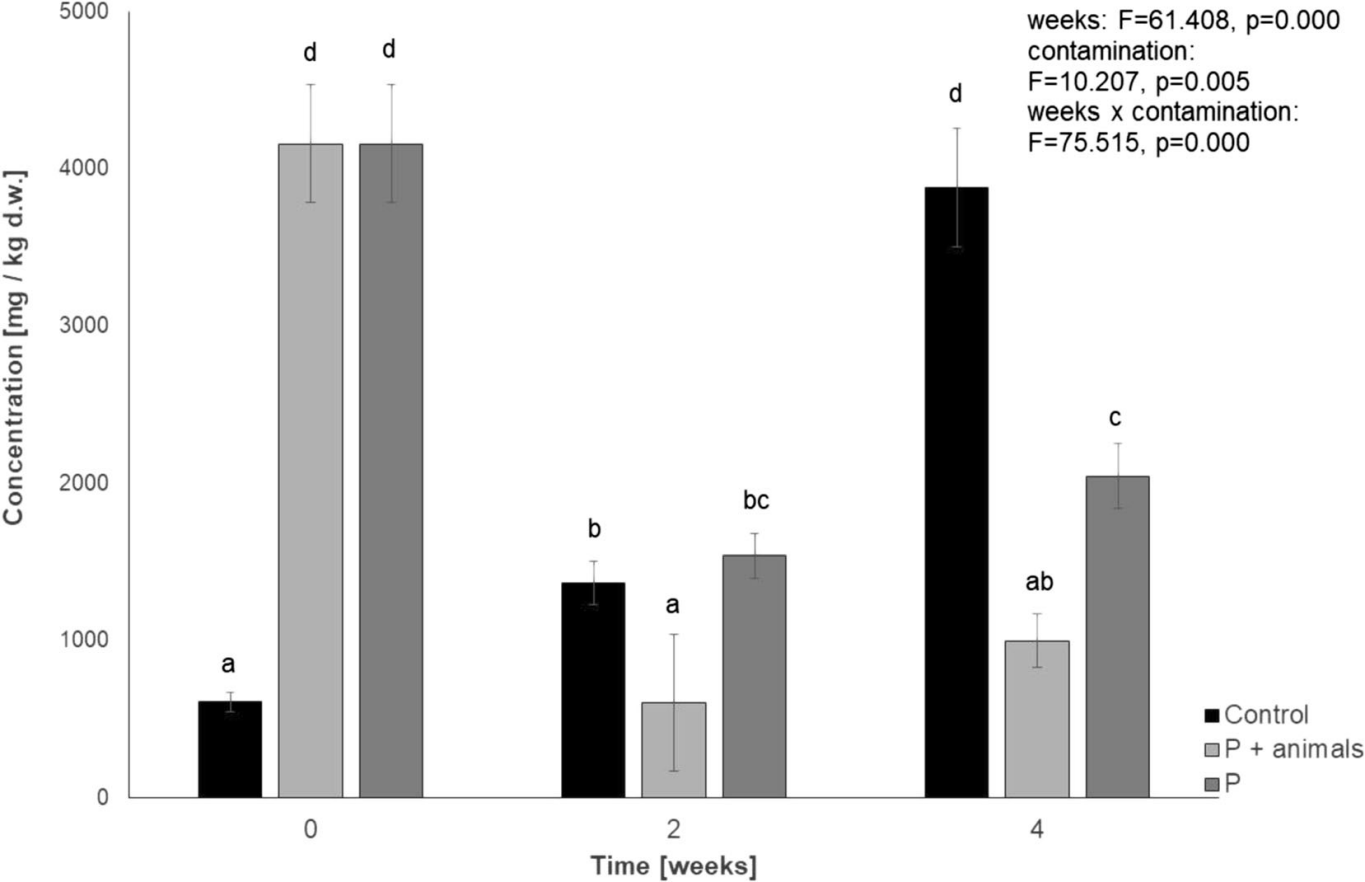
Level of total petroleum hydrocarbons in soil contaminated with petrol. (P + animals—petrol-contaminated soil with animals, *n* = 84 animals provided at the beginning of the experiment for this experimental group; P—petrol-contaminated soil without animals; control—uncontaminated soil without *P. scaber*). Means marked with the same letters do not differ significantly according to LSD test at *p* < 0.05. Factors: weeks × contamination

Table 1 shows the results of chromatographic analyses of polycyclic aromatic hydrocarbons in *P. scaber* bodies conducted in the 2nd and 4th week of the test. The results of measurements of the animals subjected to the effect of petrol were compiled in column P. It is worth noticing that in case of discussed experimental variant, PAH presence in *P. scaber* bodies was observed only after 2 weeks of the experiment duration. At the same time, in case of the experimental variant involving *Porcellio*, almost 75% depletion of oil derivatives was determined (Fig. 3), in relation to c.a. 50% loss observed in the sample without the animals. Observed high level of the decrease in TPH level may result from intensive evaporation of these compounds and the soil microbiological activity. It cannot be unanimously stated if a 25% difference between the sample inoculated and not inoculated with the animals results from their ability of metabolizing hydrocarbons.

**Table 1:** Concentration of selected PAHs in bodies of *Porcellio scaber* [μg/g wet weight of animal material] during exposure to various hydrocarbon compounds (2—after 2 weeks of experiment, 4—after 4 weeks of experiment, P—soil contaminated with petrol, DF—soil contaminated with diesel fuel, EO—soil contaminated with engine oil, nd—not detected). Presented results are average values of two independent repetitions with the calculated standard deviation

PAHs	P		DF		EO	
	2	4	2	4	2	4
Acenaphthylene	0.04 ± 0.01^a^	nd	0.02 ± 0.004^a^	nd	nd	nd
Fluorene	nd	nd	nd	0.15 ± 0.02	nd	nd
Phenanthrene	0.11 ± 0.02^a^	nd	0.07 ± 0.01^a^	0.21 ± 0.04^b^	0.07 ± 0.006^a^	nd
Anthracene	0.04 ± 0.013^a^	nd	0.22 ± 0.03^b^	0.40 ± 0.03^c^	nd	0.05 ± 0.01^a^
Pyrene	nd	nd	nd	nd	0.05 ± 0.02	nd
Benz[*a*]anthracene	0.01 ± 0.002^a^	nd	0.03 ± 0.002^a^	nd	0.13 ± 0.03^b^	nd
Chrysene	nd	nd	nd	nd	nd	0.01 ± 0.003
Benzo[*b*]fluoranthene	nd	nd	nd	nd	nd	nd
Benzo[*k*]fluoranthene	nd	nd	nd	nd	0.23 ± 0.07	nd
Benzo[*a*]pyrene	0.18 ± 0.04^b^	nd	0.01 ± 0.002^a^	nd	0.19 ± 0.03^b^	nd
Dibenz[*ah*]anthracene	nd	nd	nd	nd	nd	nd
Benzo[*ghi*]perylene	0.28 ± 0.03^b^	nd	nd	nd	0.06 ± 0.01^a^	nd

*Means in lines marked with the same letters do not differ significantly according to LSD test at *p* < 0.05

**Fig. 4:**
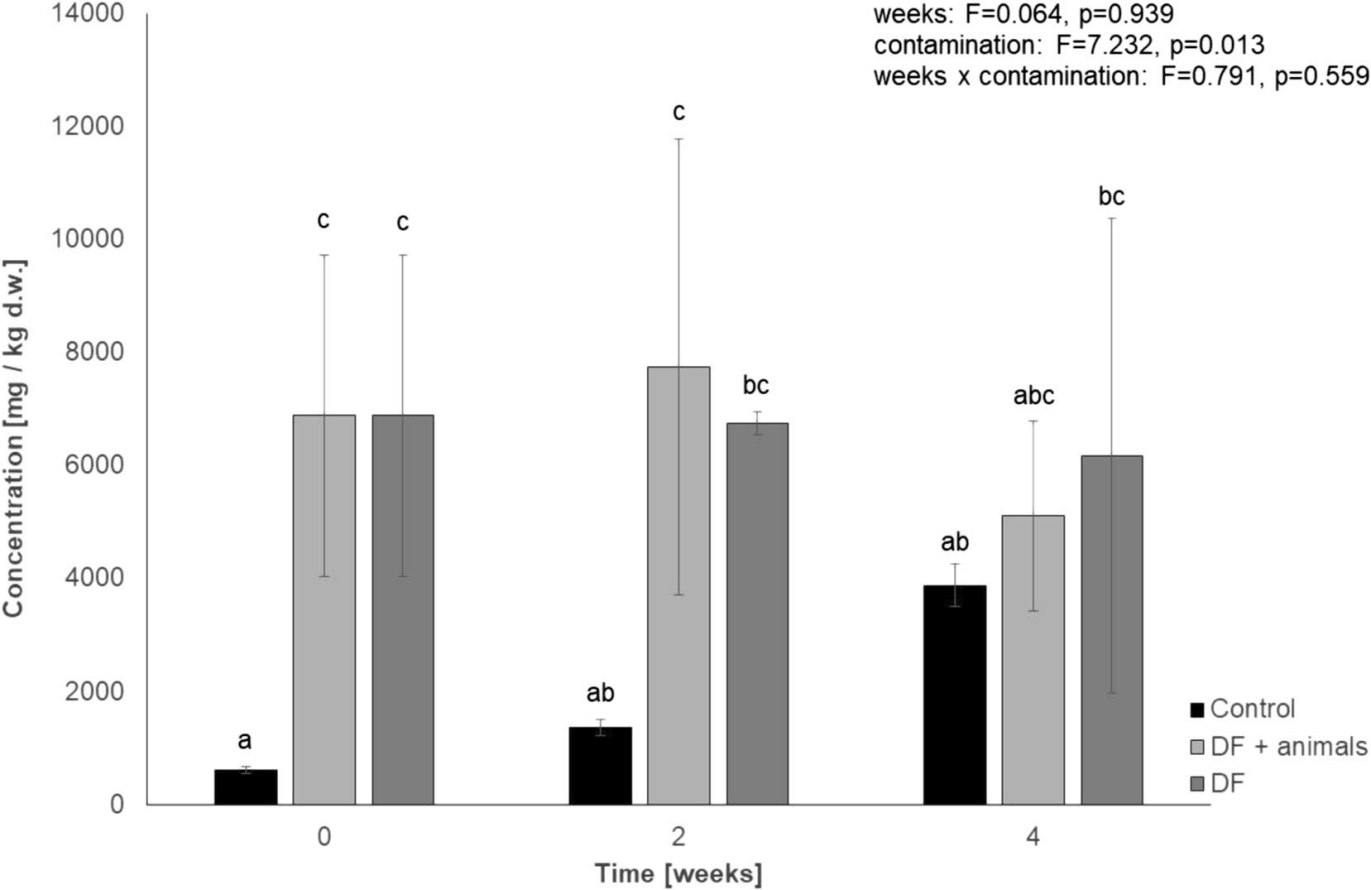
Level of total petroleum hydrocarbons in soil contaminated with diesel fuel. (DF + animals—diesel fuel–contaminated soil with animals, *n* = 84 animals provided at the beginning of the experiment for this experimental group; DF—diesel fuel–contaminated soil without animals; control—uncontaminated soil without *P. scaber*). Means marked with the same letters do not differ significantly according to LSD test at *p* < 0.05. Factors: weeks × contamination

**Fig. 5:**
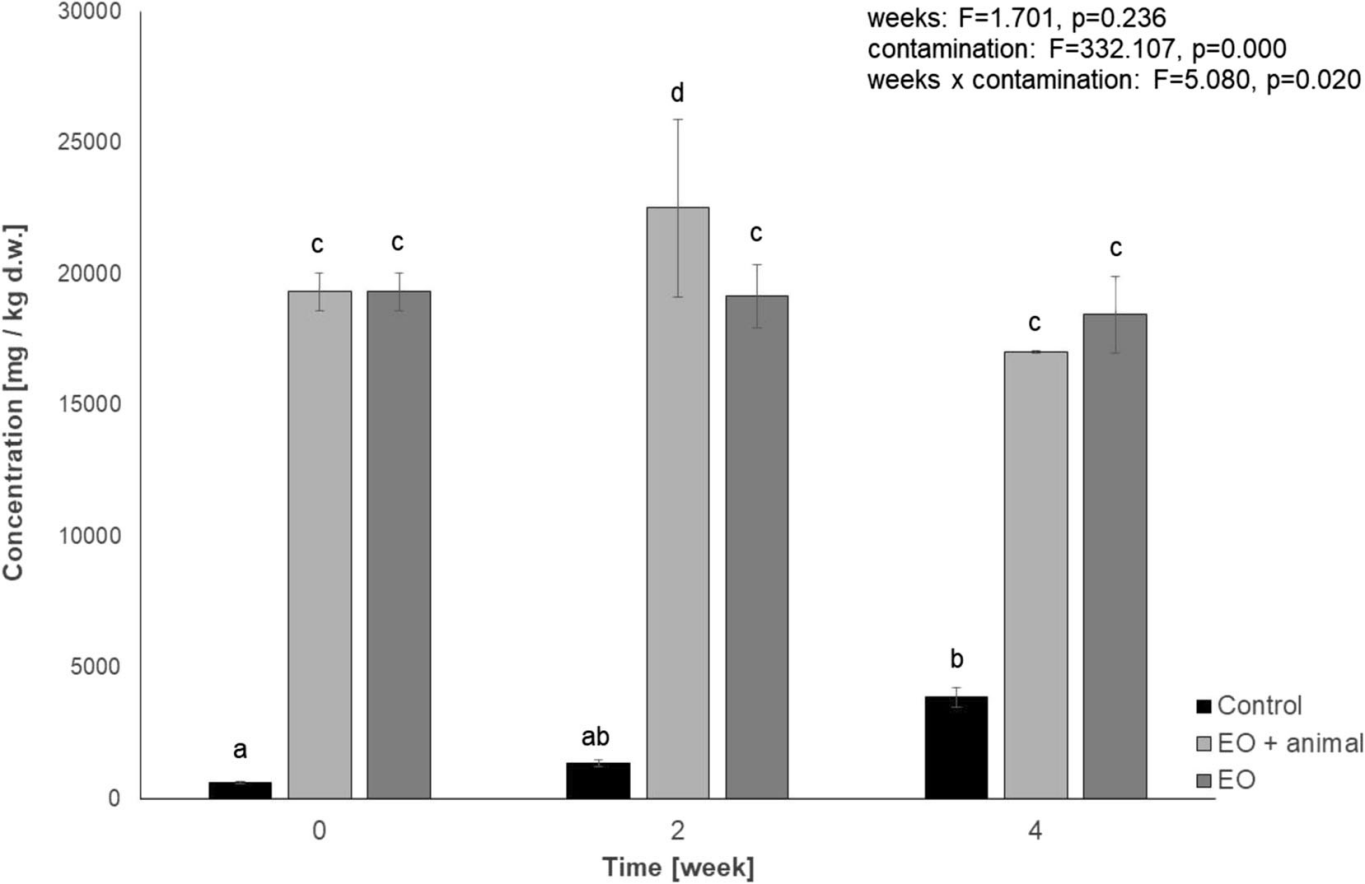
Level of total petroleum hydrocarbons in soil contaminated with engine oil. (EO + animals—engine oil–contaminated soil with animals, *n* = 84 animals provided at the beginning of the experiment for this experimental group; EO—engine oil–contaminated soil without animals; control—uncontaminated soil without *P. scaber.* Means marked with the same letters do not differ significantly according to LSD test at *p* < 0.05. Factors: weeks × contamination

Results of PAH content in the animal material from rearing on the soil contaminated with diesel fuel were compiled in column DF in Table 1. High levels of phenanthrene and anthracene, respectively, 0.07 and 0.21 μg/g of wet mass for phenanthrene and 0.22 and 0.40 μg/g of wet mass for anthracene, observed successively in the 2nd and 4th week of rearing should be noticed. Registered level of PAHs may point to a tendency for accumulation of these compounds in *P. scaber* organisms. In the EO column of Table 1, the PAH content in the tissue of *P. scaber* during rearing in engine oil–contaminated soil is shown. Despite identifying phenanthrene after 2 weeks of the experiment (0.07 μg/g of wet mass) in the bodies of animals, no evidence of its bioaccumulation has been noted after 4 weeks of rearing. After the 2nd week of the experiment, a relatively high level of benzo[*a*]pyrene and benzo[*k*]fluoranthene was detected (0.19 and 0.23 μg/g of wet mass, respectively); however, none of these hydrocarbons was identified after ending of the experiment. It is likely that in *P. scaber* tissues biotransformation of mentioned polyaromatic hydrocarbons occurs. During the experiment, we did not identify any intermediate compounds that could indicate direct metabolism of hydrocarbons by animals. Due to the fact that hydrocarbons were isolated from whole animals, we were unable to distinguish whether they were inside the body or if they were adsorbed to the outer surface of the shell. Figures 4 and 5 show results of a change of the total hydrocarbons when rearing was conducted on soils contaminated with diesel fuel and engine oil. No influence of the animals presence on the TPH degradation rate was detected.

The level of benzo[*a*]pyrene (the 2nd week of the experiment duration, respectively, *P*—0.18, DF—0.01, and OE—0.19 μg/g of wet mass) is worth noticing. Van Brummelen and Van Straalen (1996) determined benzo[*a*]pyrene in *P. scaber* bodies on an average level of 2.5 μg of the xenobiotic/g d.m., which persisted for almost 50 days of the experiment. However, it should be mentioned that the researchers supplied food contaminated by this hydrocarbon to the insects, whereas in the presented experimental design the animals were fed with unpolluted food. A relatively low level of hydrocarbon in *P. scaber* bodies may confirm that the animals are exposed to PAHs mainly through their food intake. On the other hand, they are less susceptible to these xenobiotic penetrations through integuments. In most cases, pyrene content in the bodies of examined animals was undetectable (Table 1). It was assessed only in the animals cultured on the soil contaminated with used engine oil after 2 weeks of exposure (0.05 μg/g of wet mass). Research on the ability for PAH detoxification in *P. scaber* revealed an ability for pyrene metabolizing in the hepatopancrea and gut through pyrene oxidation to 1-hydroxypyrene, 1-hydroxypyrene sulphate, hydroxypyrene glycoside, and three unidentified 1-hydroxypyrene conjugates (Stroomberg et al. 1999). Later investigations conducted by the authors (Stroomberg et al. 2004b) revealed that *P. scaber* shows a higher efficiency in pyrene metabolism than *Eisenia andrei* earthworm, whereas one of the previously unrecognized metabolites is pyrene-1-O-(6”-O-malonyl)glucoside. Research conducted by Knecht et al. (2001) also points to considerable abilities of Isopoda for PAH biotransformation. The authors determined a very low level of PAHs remains in *P. scaber* and *O. asellus* bodies, despite their being fed with food polluted with benzo[*a*]pyrene and 3-methylcholanthrene.

## Conclusions

1. *Porcellio scaber* reveals a considerable resistance in a short contact with petroleum derived substances, evidenced as high survivability and undisturbed increase in body mass.
2. No positive correlation was observed between TPH depletion in the soils contaminated with PDSs and *P. scaber* presence during 4 weeks of rearing.
3. PAH level in *P. scaber* bodies was generally very low, which may confirm the thesis about considerable abilities of isopods for biotransformation of these pollutants and low susceptibility to these xenobiotic penetration through integuments. However, a tendency for accumulation for phenanthrene and anthracene in conditions of soil polluted with diesel fuel was observed.
